# Quantitative detection of myocardial ischaemia by stress echocardiography; a comparison with SPECT

**DOI:** 10.1186/1476-7120-7-28

**Published:** 2009-06-18

**Authors:** Petri Gudmundsson, Kambiz Shahgaldi, Reidar Winter, Magnus Dencker, Mariusz Kitlinski, Ola Thorsson, Ronnie B Willenheimer, Lennart Ljunggren

**Affiliations:** 1 Department of Biomedical Laboratory Science, Malmö University, Malmö, Sweden; 2 Department of Cardiology, Karolinska University Hospital Huddinge, Stockholm, Sweden; 3 Department of Clinical Physiology, Karolinska University Hospital Huddinge, Stockholm, Sweden; 4 Department of Clinical Physiology, Lund University, Malmö University Hospital, Malmö, Sweden; 5 Department of Cardiology, Lund University, Malmö University Hospital, Malmö, Sweden; 6 Department of Clinical Sciences, Medicine/Cardiology, Lund University, Malmö University Hospital, Malmö, Sweden; 7 Heart Health Group, Malmö, Sweden

## Abstract

**Aims:**

Real-time perfusion (RTP) adenosine stress echocardiography (ASE) can be used to visually evaluate myocardial ischaemia. The RTP power modulation technique angio-mode (AM), provides images for off-line perfusion quantification using Qontrast^® ^software, generating values of peak signal intensity (A), myocardial blood flow velocity (β) and myocardial blood flow (Axβ). By comparing rest and stress values, their respective reserve values (A-r, β-r, Axβ-r) are generated. We evaluated myocardial ischaemia by RTP-ASE Qontrast^® ^quantification, compared to visual perfusion evaluation with ^99m^Tc-tetrofosmin single-photon emission computed tomography (SPECT).

**Methods and Results:**

Patients admitted to SPECT underwent RTP-ASE (SONOS 5500) using AM during Sonovue^® ^infusion, before and throughout adenosine stress, also used for SPECT. Visual myocardial perfusion and wall motion analysis, and Qontrast^® ^quantification, were blindly compared to one another and to SPECT, at different time points off-line.

We analyzed 201 coronary territories (left anterior descendent [LAD], left circumflex [LCx] and right coronary [RCA] artery territories) in 67 patients. SPECT showed ischaemia in 18 patients and 19 territories. Receiver operator characteristics and kappa values showed significant agreement with SPECT only for β-r and Axβ-r in all segments: area under the curve 0.678 and 0.665; P < 0.001 and < 0.01, respectively. The closest agreements were seen in the LAD territory: kappa 0.442 for both β-r and Axβ-r; P < 0.01. Visual evaluation of ischaemia showed good agreement with SPECT: accuracy 93%; kappa 0.67; P < 0.001; without non-interpretable territories.

**Conclusion:**

In this agreement study with SPECT, RTP-ASE Qontrast^® ^quantification of myocardial ischaemia was less accurate and less feasible than visual evaluation and needs further development to be clinically useful.

## Introduction

In low risk patients with suspected myocardial ischaemia, evaluation of ischaemia is generally recommended for optimal care and treatment [1,2]. Exercise ECG is considered the first line technique for assessment of ischaemia, whereas single-photon emission computed tomography (SPECT) or dobutamine atropine stress echocardiography (DSE) are suggested when exercise ECG are non-diagnostic or non-interpretable [3]. Both SPECT and DSE are well established and more accurate methods than exercise ECG [4-7], although more expensive. Adenosine stress echocardiography (ASE) can also be used for ischaemia evaluation, but demands evaluation of myocardial perfusion to reach similar accuracy for detecting ischaemia and can not solely rely on wall motion assessment [8,9]. The use of second generation myocardial contrast agents enables real time myocardial perfusion (RTP) echocardiography. RTP combined with ASE has shown promising results in evaluating myocardial ischaemia in different patient populations and settings [10-18]. RTP has one possible advantage comparing to all three mentioned techniques; the ability to follow replenishment of myocardial perfusion in real-time. Therefore, RTP has the ability to compare myocardial perfusion and replenishment rate at rest and stress, which could add valuable information and perhaps increase the sensitivity of myocardial ischaemia detection. One drawback is the subjectivity of visual myocardial perfusion evaluation by echocardiography, which demands experienced interpreters and limits the use of RTP-ASE. Techniques for objective quantification of myocardial perfusion in echocardiography are evolving and software programs are now commercially available. The quantitative techniques have shown promising results in animal experiments [19,20] and in humans [21-25]. However, there are few studies from clinical settings and most of these have been done with different software. If a quantitative echocardiographic technique were to show equivalent results to SPECT in detecting myocardial ischaemia, it could be an alternative method, more available and without radiation compared to SPECT, more tolerable and swifter than DSE, and more accurate than exercise ECG.

Qontrast^® ^(AMID^®^, Roma, Italy; Bracco™, Milan, Italy) is a recently developed and commercially available software, with algorithms that automatically follow the left myocardium contours throughout the cardiac cycle and throughout the replenishment period of the RTP image loop. Qontrast^® ^may provide a practical way to quantify myocardial perfusion by contrast echocardiography, and has shown promising initial results in both animals and patients with acute myocardial infarction [20,26]. However, it has not yet been investigated in patients with suspected stable myocardial ischaemia.

The aim of the present study was to examine if RTP-ASE Qontrast^® ^quantification can be used to correctly evaluate myocardial ischaemia in patients with known or suspected stable coronary artery disease, as compared with visual evaluation of ischaemia by RTP-ASE, as well as with SPECT.

## Methods

### Patient population

We prospectively asked 69 randomly selected patients, without prior knowledge of acoustic windows, admitted to adenosine SPECT evaluation of known or suspected stable coronary artery disease, to participate in the study. Part of the study population has been presented previously [18]. Two of the included patients had visually non-interpretable echocardiography images and were, therefore, excluded from the study. The institutional ethics committee of the Lund University, Sweden, approved the study. Written informed consent was obtained from all participating patients.

### Study protocol

#### Myocardial contrast echocardiography

The echocardiographic equipment used was a Sonos 5500 (Philips, Andover, Massachusetts, USA) with S3 probe and RTP using the power modulation angio-mode. Patients were examined in a left lateral recumbent position. The second-generation contrast agent Sonovue^® ^was infused in the left decubital vein using an infusion pump dedicated for this purpose (VueJect^® ^Esaote, Genova, Italy; Bracco™, Milan, Italy), which automatically rotates the syringe to prevent sedimentation. The infusion rate of Sonovue was set between 1.0 and 1.3 ml/min [27]. Adenosine and echo contrast were infused in the same peripheral venous catheter, using a separate infusion pump through a three-way tap. Adenosine was given at an infusion rate of 100 μg/kg/min during one minute, after which the infusion rate was increased to 140 μg/kg/min.

All 67 patients underwent RTP imaging (mechanical index = 0.1) during infusion of echo contrast, at rest and after a minimum of one minute of hyperaemia during adenosine stress (at 140 μg/kg/min). Image acquisition was started after at least one minute of Sonovue infusion. RTP image loops containing 8–10 heartbeats were collected from the parasternal long-axis and apical four- and two-chamber views, respectively. At the beginning of each loop a destruction impulse of 10 high mechanical index frames (mechanical index = 1.5) were given to destroy all contrast micro bubbles in the myocardium [28].

During RTP the angio-mode gain was set between 60 and 70%, depending on what was suitable for the individual patient as judged by a visual on-line assessment, and 2D greyscale gain was set at zero. Focus was set close to the base of the left ventricle. All images were stored digitally for later off-line analysis.

#### Qontrast^® ^evaluation

**Figure 1:**
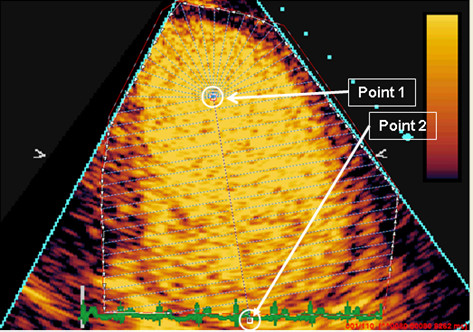
**Manually placed points to enable an automatic outline of the complete left ventricle, including both cavity and myocardium, before automated Qontrast^® ^perfusion analysis**.

Qontrast^® ^was used to produce parametric images of contrast replenishment values for the RTP loops collected from both rest and stress. Two points were manually placed in the left ventricular cavity of the perfusion images. The first point was placed in the centre of the cavity where the apex "half-circle" ends, i.e. approximately two thirds from the base of ventricle, where it was always inside the cavity (never in the myocardium) during the complete loop. The point was placed in a cavity area that was fully opacified directly after the destruction impulse in the beginning of the loop, as well as throughout the entire RTP-loop, since this formed the basis of the maximum image contrast intensity reference-point. Any isolated frames not fulfilling these criteria were excluded from analysis. The second point was placed at the base of the ventricle, enabling the software to automatically outline the complete left ventricle, including both cavity and myocardium, with dotted "M-mode" lines crossing perpendicular through the myocardial wall (Figure 1).

The first frame was selected to be the one directly after destruction impulse frames.

Automatic perfusion analysis was then started. Qontrast^® ^uses an advanced image processing technique that recognizes the coherence of the dynamic image sequence in a space-time domain. The technique enables tracking of the myocardial pixel movement throughout the cardiac cycle and the entire RTP-loop. This increases the accuracy of the perfusion evaluation compared to triggered imaging, due to the higher number of quantifiable frames.

Three parametric images were then automatically generated from the perfusion analysis, displaying either the peak signal intensity (A), myocardial blood flow velocity (β) or myocardial blood flow (Axβ). These were generated for each pixel, from the replenishment curve of each pixel, according to the replenishment curve A = A(1-e^-βt^) [29]. These parametric images were generated from RTP images in apical four- and two-chamber and parasternal long-axis view, at rest and stress, respectively.

**Figure 2:**
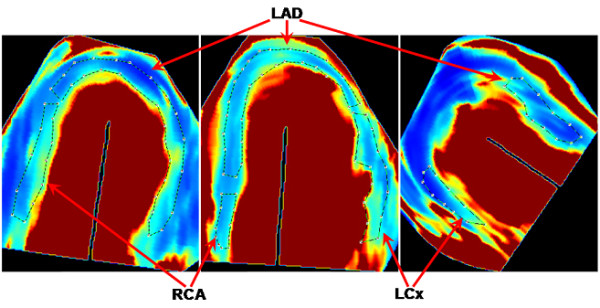
**Tracings of coronary territories of interest in four-chamber (middle), two-chamber (left) and long-axis (right) views**. LAD, left anterior descending; LCx, left circumflex; RCA, right coronary artery.

To acquire quantitative values of A, β and Axβ, region of interests were manually traced both at rest and stress, corresponding to the distribution territories of the three main coronary arteries; left anterior descending (LAD), left circumflex (LCx) and right coronary artery (RCA) (Figure 2). Since earlier studies indicated that β is the most sensitive quantitative parameter [24,30], special care was taken that the tracing would align correctly in the parametric β image, avoiding red areas. These either correspond to contrast in the left ventricular cavity, are due to perfusion artefacts originating from main coronary arteries, or are caused by mathematically generated high β-values due to very low A-values, which predominantly occur in rest images where the A-values are low for physiological reasons.

Comparing A, β and Axβ values at rest and stress (hyperaemia), the corresponding reserve values (A-r; β-r and Axβ-r) were derived by dividing the stress value with the matching rest value, thus resembling invasive measurement of coronary flow reserve. Accordingly, this generated three A-r, β-r, and Axβ-r values from the LAD territory, originating from the three different echocardiographic views (four-chamber, two-chamber, and long-axis views), two reserve values each from the LCx territory (four-chamber and long-axis views) and two from the RCA territory (four- and two-chamber views). The lowest A-r, β-r and Axβ-r value from any of the views corresponding to a coronary territory was selected for the ischaemia comparison with SPECT, since the lowest reserve value should originate from the most ischaemic or the least perfused territory.

#### RTP-ASE visual ischaemia interpretation

Two separate visual image interpretation were performed with off-line analysis of myocardial perfusion and wall motion at RTP-ASE, using the EnConcert Image Diagnosis Application (Philips, Andover, Massachusetts, USA). The first visual analysis was a combined analysis of perfusion and wall motion. The second visual analysis, performed separately, was an analysis excluding wall motion to estimate the value of sole perfusion analysis. Visual and quantitative perfusion analyses were done on separate occasions, blinded to one another and blinded to the result of SPECT. Each segment of the left ventricular myocardium was attributed to the same coronary vessel territory at all analyses. Myocardial ischaemia was visually evaluated comparing rest and stress images, using both perfusion and wall motion analysis in a complementary manner. A visually detected perfusion defect during stress was used as the principal marker of ischaemia. Thus, a myocardial segment was considered ischaemic if perfusion was impaired in the stress images, compared to the rest images [8]. Perfusion defects were analyzed during the first four beats after the destruction impulse at rest and after two beats at peak stress. Wall motion was used in addition to reveal perfusion defect artefacts at rest and to evaluate segments with suspected perfusion artefacts at stress. Since wall motion should not be normal if a segment has a true perfusion defect at rest, a perfusion defect at rest was considered to be an artefact when wall motion was normal in that segment. A perfusion defect at peak stress was considered to be an artefact if there was a suspicion of a perfusion artefact, such as lateral or anterior shadowing from ribs or lungs, or basal segments shadowed by contrast. In such segments, the ischaemic evaluation was based on wall motion analysis alone. If wall motion decreased at stress compared to rest images, the segment was considered ischaemic. Since perfusion can be decreased without a decrease in wall motion at ASE, the use of solitary wall motion analysis in segments with perfusion artefacts might decrease the sensitivity with regard to ischaemia. However, this complementary use of wall motion analysis increases the number of interpretable segments without negatively affecting specificity [12,18]. If there was disagreement between readers with regard to one segment, this particular segment was re-assessed in a joint reading until consensus was reached.

#### SPECT

The rest and stress studies were performed using a 2-day protocol, starting with injection of 600 MBq ^99m^Tc-tetrofosmin at stress. Stress was simultaneous with the RTP-ASE. Normal findings at stress were not followed by a rest study [31,32]. Pathological stress studies were followed by a rest study with injection of 800 MBq ^99m^Tc-tetrofosmin. Patients who had cardiac medications, which could interfere with the stress test, were informed to have their medication interrupted prior to the stress test. The decision whether to interrupt the drug administration was at the discretion of the referring physician. A five-minute adenosine infusion protocol was used. Starting the infusion with 100 μg/ml/min of adenosine for 1 minute, the dose was then increased to 140 μg/ml/min for two minutes before injecting ^99m^Tc-tetrofosmin. Infusion of adenosine was continued for 2 min after the injection of ^99m^Tc-tetrofosmin. The scintigraphic data were acquired one hour after the end of the stress test, using continuous SPECT over 180 degree elliptical rotation from the 45 degree right anterior oblique position, with a dual-head gamma camera (Siemens AG Medical Solutions, Erlangen, Germany). Low energy high-resolution collimator and a zoom factor of 1.0 were used. We obtained 64 projections in a 128 × 128 matrix, with an acquisition time of 20 s per projection. Tomographic reconstruction and calculation of short axis slice images were performed using Siemens software. A two-dimensional Butterworth pre-reconstruction filter was used with critical frequency of 0.35, order 5. For each patient, the same sets of short axis slices were then processed with an automatic software package (4D-MSPECT) on a Siemens e.soft workstation. The software package defined apex and base and generated, coronal, longitudinal, sagital tomographic slices as well as polar maps with schematic map of the territories of the main coronary arteries used for scoring. Radiotracer uptake of the vascular segments were scored visually and stress images were compared with rest images regarding ischaemia and infarct. The specialist in nuclear medicine who performed the scoring was blinded to the results of the RTP analysis.

### Statistical analysis

Method of reference for the ischaemia evaluation in the study was the presence or absence of reversible ischaemia at the SPECT examination. Continuous variables are expressed as mean ± 1SD and as percent. P < 0.05 denoted significance. Receiver operating characteristic (ROC) curves were used to examine and compare predictive ability of different parametric variables, by calculating sensitivity, specificity, accuracy, positive and negative predictive values (PPV, NPV) and area under the curve (AUC). Unpaired t-test was used to test for difference between patients. For intra-assay variability of quantitative measurements of A and β, coefficient of variation was used.

## Results

Clinical data were retrieved form patients records. Patient characteristics are presented in Table 1. Adenosine infusion had minor but significant effects on both heart rate and blood pressure, where heart rate increased from 72 ± 14 to 82 ± 14 (p < 0.001), systolic blood pressure decreased from 133 ± 20 to 127 ± 20 (p < 0.001) and pulse-pressure product increased from 9.65 ± 2.32 k to 10.44 ± 2.57 k (p < 0.001). Only minor side-effects occurred and no stress-test had to be interrupted due to side-effects. At SPECT 18 patients (27%) were ischaemic with a total of 19 ischaemic territories; 10 LAD territories, 5 LCx territories and 4 RCA territories.

**Table 1:** Patient characteristics (n = 67 unless otherwise noted).

Age	68 (± 10)
Male	33 %
LVEF at rest	54 (± 11) %
Previous AMI	40 %
Previous PCI	19 %
Previous CABG	13 %
Heart failure	13 %
Hypertension	48 %
Valvular surgery	0 %
Beta-blocker	57 %
ACE inhibitor	28 %
ARB	12 %
Nitro-glycerine (short acting)	57 %
Nitrates (long acting)	25 %
Diuretics	27 %
Calcium blocker	18 %
Sinus rhythm	93 %
Dilated left ventricle	13 %
Dilated left atrium (n = 43)	33 %
Significant valvular disease (n = 43)	7 %
Regional WMA/PD at rest	60 %

LVEF, left ventricular ejection fraction; AMI, acute myocardial infarction; PCI, percutaneous coronary intervention, CABG, coronary artery bypass grafting; ACE, angiotensin converting enzyme; ARB, angiotensin-receptor blocker, WMA, wall motion abnormality; PD, perfusion defect.

Of the 201 coronary distribution territories, 28 (14%) could not be analyzed due to perfusion artefacts according to the visual perfusion analysis. These territories were still evaluated in the visual perfusion analysis with combined wall motion evaluation, since wall motion still could be analyzed in these territories. In the quantitative analysis two more territories were considered non-interpretable using the Qontrast^® ^software due to low parametric image quality, which made it too difficult to differentiate the left ventricular myocardium from the cavity. A summary of non-interpretable territories is presented in Table 2. In Table 3 the different results from both quantitative and visual interpretations are summarized. Quantitative reserve parameters β-r and Axβ-r showed significant AUC and kappa in all coronary territories. All quantitative reserve parameters expressed significant kappa values in the LAD coronary territory. Both visual analyses demonstrated higher kappa values and accuracy than any quantitative parameter.

**Table 2:** Non interpretable coronary territories for Qontrast^® ^quantification and visual interpretation with complementary wall motion analysis (Vis 1) and sole perfusion interpretation (Vis 2).

	All Territories (n = 201)	LAD (n = 67)	LCx (n = 67)	RCA (n = 67)	Patient (n = 67)
Qontrast^® ^(%)	30 (15)	12 (18)	16 (24)	2 (3)	15 (22)
Vis 1 (%)	0	0	0	0	0
Vis 2 (%)	28 (14)	11 (16)	16 (24)	1 (1)	22 (33)

**Table 3:** Results for the respective quantitative variables and visual interpretations (QV), A-reserve, (A-r), β-reserve, (β-r) and Axβ-reserve (Axβ-r), visual RTP-ASE interpretation with complementary wall motion (Vis 1) and with sole perfusion interpretation (Vis 2).

QV	Coronary Territory	Acc (%)	Sens (%)	Spec (%)	PPV (%)	NPV (%)	kappa	AUC
A	All	*43*	*39*	*75*	*11*	*94*	*NS*	*0.529 NS*
**A**	**LAD**	**67**	**66**	**75**	**27**	**94**	0.237 *	*0.705 NS*
A	LCx	*29*	*28*	*50*	*6*	*87*	*NS*	*0.229 NS*
A	RCA	*32*	*28*	*100*	*8*	*100*	*NS*	*0.518 NS*
**β**	**All**	**80**	**83**	**56**	**25**	**95**	0.249 ***	0.678 *
**β**	**LAD**	**82**	**83**	**75**	**43**	**95**	0.442 **	0.773 *
β	LCx	*82*	*94*	*25*	*25*	*94*	*NS*	*0.590 NS*
β	RCA	*72*	*74*	*50*	*11*	*96*	*NS*	*0.666 NS*
**Axβ**	**All**	**75**	**77**	**63**	**22**	**95**	0.213 **	0.665 *
**Axβ**	**LAD**	**82**	**83**	**75**	**43**	**95**	0.442 **	0.818 **
Axβ	LCx	*90*	*96*	*25*	*33*	*94*	*NS*	*0.404 NS*
Axβ	RCA	*58*	*57*	*75*	*10*	*97*	*NS*	*0.680 NS*
A	Patient wise	*35*	*11*	*100*	*29*	*11*	*NS*	*NA*
**β**	**Patient wise**	**60**	**53**	**79**	**38**	**87**	0.233 *	*NA*
Axβ	Patient wise	*47*	*38*	*73*	*31*	*79*	*NS*	*NA*
**Vis 1**	**All**	**93**	**90**	**93**	**59**	**99**	0.67 ***	*NA*
**Vis 1**	**LAD**	**87**	**90**	**86**	**53**	**98**	0.59 ***	*NA*
**Vis 1**	**LCx**	**94**	**100**	**94**	**56**	**100**	0.68 ***	*NA*
**Vis 1**	**RCA**	**96**	**75**	**100**	**100**	**98**	0.85 ***	*NA*
**Vis 1**	**Patient wise**	**90**	**89**	**90**	**76**	**96**	0.75 ***	*NA*
**Vis 2**	**All**	**92**	**94**	**92**	**55**	**99**	0.67 ***	*NA*
**Vis 2**	**LAD**	**86**	**100**	**83**	**53**	**100**	0.61 ***	*NA*
**Vis 2**	**LCx**	**94**	**100**	**94**	**57**	**100**	0.68 ***	*NA*
**Vis 2**	**RCA**	**95**	**75**	**97**	**60**	**98**	0.64 ***	*NA*
**Vis 2**	**Patient wise**	**89**	**91**	**88**	**71**	**97**	0.73 ***	*NA*

A, peak signal intensity; β, myocardial blood flow velocity; Axβ, myocardial blood flow; Acc, accuracy; Sens, sensitivity; Spec, specificity; PPV, positive predictive value; NPV, negative predictive value; AUC, area under the curve; * = p < 0.05; ** = p < 0.01; *** = p < 0.001; NS, not significant; NA, not applicable. Bold indicates significance and italics indicates not significant or not applicable.

**Figure 3:**
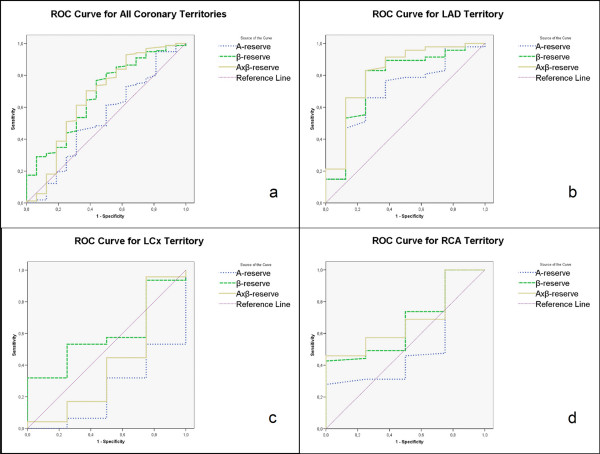
**a-d. Receiver operator characteristics curves of the quantitative perfusion measurements A-, β- and Axβ- reserve in all coronary territories (a), LAD territory (b), LCx territory (c) and RCA territory (d), as compared with ischaemia at SPECT**. LAD, left anterior descending; LCx, left circumflex; RCA, right coronary artery; A-r, peak signal intensity reserve; β-r, myocardial blood flow velocity reserve; Axβ-r, myocardial blood flow reserve.

**Figure 4:**
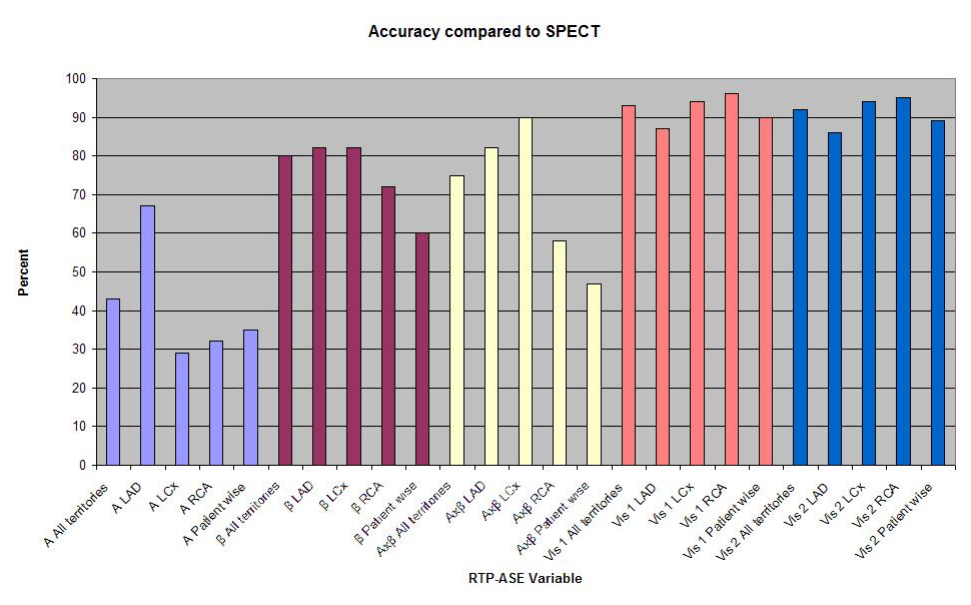
**A graph of accuracy between different modalities of RTP-ASE versus SPECT**. Left anterior descending (LAD), left circumflex (LCx) and right coronary artery (RCA). Peak signal intensity (A), myocardial blood flow velocity (β), myocardial blood flow (Axβ).

From the ROC curves (Figure 3a-d) AUC is visualized and levels of sensitivity at different specificity values can be estimated. Despite the non-significant AUC for all reserve variables in the LCx and RCA territories, there is a notable level of sensitivity at preserved 100% specificity for β-r in both territories, and for Axβ-r in the RCA territory. Figure 4 represents a graph of accuracy between different modalities of RTP-ASE versus SPECT. When dividing the patient population into those without and those with ischemia at SPECT, there were significant differences for A-r, β-r and Axβ-r in the LAD territory and for β-r in the all-territory analysis. All variables and territory differences are given in Table 4.

**Table 4:** t-test for quantitative variables (QV) peak signal intensity (A), myocardial blood flow velocity (β) and myocardial blood flow (Axβ) at rest, stress and their respective reserves, if no ischaemia or ischaemia at SPECT.

		No Ischaemia at SPECT			Ischaemia at SPECT		
QV	CA	Rest	Stress	Reserve	Rest	Stress	Reserve

A	All	46.9 ± 14.6	55.7 ± 10.1	1.13 ± 0.60	44.3 ± 15.3	52.0 ± 9.6	1.33 ± 1.26
A	LAD	48.7 ± 9.7	56.7 ± 11.7	1.05 ± 0.32	51.2 ± 7.9	51.3 ± 4.9	0.86 ± 0.20 *
A	LCx	37.1 ± 14.1	53.8 ± 6.5	1.48 ± 0.91	26.0 ± 12.4	46.7 ± 1.3 **	2.78 ± 2.00
A	RCA	54.9 ± 13.3	56.3 ± 10.9	0.92 ± 0.28	51.5 ± 13.5	57.2 ± 17.0	0.82 ± 0.34

β	All	1.12 ± 0.41	4.7 ± 3.3	2.52 ± 1.98	1.26 ± 0.38	3.2 ± 1.6 **	1.55 ± 0.87 *
β	LAD	1.15 ± 0.36	3.8 ± 1.6	1.77 ± 0.75	1.28 ± 0.25	2.7 ± 1.3	1.20 ± 0.59 *
β	LCx	1.16 ± 0.47	6.4 ± 4.3	3.58 ± 2.74	1.04 ± 0.26	3.9 ± 2.3	2.39 ± 1.14
β	RCA	1.04 ± 0.40	4.1 ± 2.8	2.28 ± 1.59	1.51 ± 0.64	3.4 ± 1.8	1.41 ± 0.65 *

Axβ	All	55.7 ± 32.4	261 ± 18	3.54 ± 4.60	59.6 ± 31.5	172 ± 100 **	2.87 ± 4.13
Axβ	LAD	60.2 ± 26.9	222 ± 118	2.21 ± 1.38	68.0 ± 17.2	138 ± 58 **	1.11 ± 0.71 *
Axβ	LCx	47.0 ± 34.5	346 ± 235	6.20 ± 7.24	28.5 ± 16.7	187 ± 111	7.91 ± 6.18
Axβ	RCA	60.2 ± 33.7	232 ± 166	2.53 ± 2.17	79.7 ± 45.8	222 ± 147	1.35 ± 0.85 *

CT, coronary territory; *, p < 0.05; **, p < 0.01 for level of significant difference between no ischaemia and ischaemia at SPECT.

Intra-observer variability for A and β was assessed by a second blinded reading of 10 randomly selected patients from the study. Variability was 6.0% and 18% for A and β, respectively. Visual inter- and intra-observer variability is presented in Table 5.

**Table 5:** Agreement of visual myocardial contrast echocardiography ischaemia interpretation (n = 33).

	Total	LAD	LCx	RCA
Inter-observer (%) Kappa	91 0.72***	88 0.73***	97 0.90***	88 0.28 NS
Intra-observer (%) Kappa	94 0.76***	94 0.84***	91 0.62***	97 0.78***

LAD, Left anterior descending coronary artery; LCx, Left Circumflex artery; RCA, Right posterior descending coronary artery; **, p < 0.01; ***, p < 0.001, NS, not significant.

## Discussion

This agreement study indicates that Qontrast^® ^could possibly be used for quantitative measurements of myocardial ischaemia from RTP-ASE acquired images, but only in the LAD territory. All three quantitative parameters, A-r, β-r and Axβ-r showed significant agreement with SPECT in the LAD territory. Only β-r and Axβ-r showed significant agreement with SPECT in the all-territory analysis, but in the analyses by the specific coronary territories they only showed significant agreement with SPECT in the LAD territory. Qontrast^® ^provides consistently high negative predictive values and reasonably high specificity at present cut off values, which is of importance for ruling out ischaemia. Sensitivity and positive predictive value could perhaps have been higher if the segments analyzed had been more detailed, i.e. following a 17 segment model. However, then there probably would have been a greater anatomical mismatch between echocardiography and SPECT. Visual evaluation of myocardial ischaemia showed better results than automated quantification using Qontrast^®^. The visual analysis using a combination of perfusion and wall motion to increase the number of interpretable segments, as well as the sole perfusion analysis, both showed excellent agreement with SPECT, which has been suggested previously [10,11,33,34]. It should be noted, however, that adenosine is a suboptimal stressor for wall motion analysis only [3].

There are of course differences between RTP-ASE and SPECT. The spatial resolution of echocardiography is higher than with SPECT, which might suggest missed minor ischaemic territories at SPECT. However, it is known that a SPECT image without signs of ischaemia is associated with few cardiac events [7]. Also, replenishment curves are not possible to produce using SPECT imaging, which might lead to differences between the two methods tested in the present study. The differences could have been partly elucidated if coronary angiography had been performed, and the lack of such examination. Furthermore, the fact that coronary angiography was not available in these patients prohibit us from separating perfusion defects caused by macro-vascular or by micro-vascular disease this might be considered a limitation of the study. However, both SPECT and RTP-ASE assess myocardial perfusion, whereas coronary angiography presents a morphological image, which is the main reason why SPECT was chosen as reference method. A major disadvantage of the quantitative detection of ischaemia, compared with visual interpretation, is the much lower feasibility, where the present study shows nearly 20% non-interpretable segments using Qontrast^®^. Visual interpretation with complementary wall motion analysis could be performed in all of the included patients. The study findings only apply to patient groups with suspected myocardial ischaemia and, for example, not to patient populations with acute coronary syndrome.

The results indicate that the quantification of myocardial ischaemia is still in need of improvement to be clinically useful, which is in line with previous studies [24,30]. The poor results are probably due to the high level of software automatic tissue recognition. However, this is expected to improve in the future, when both echocardiographic image quality and the algorithms for tissue or contrast recognition are expected to improve, leading to higher signal to noise ratio[35]. So far in echocardiography, human brain capacity and experience seem to beat the computer in perfusion contrast echocardiography. Still, a combination of visual and software-based analysis might be of interest [23,36]. Moreover, the justification of the cost of echo contrast and its real clinical additive value needs to be conclusively proven.

### Contrast safety

The U.S. Food and Drug Administration issued on October the 10th 2007 a "black box" warning for perflutren-containing contrast agents, which caused considerable controversies within the echocardiography community [37]. This warning was later relaxed [38]. Three recent large retrospective studies have disputed the suggestion that using the current generation echo contrast would pose a hazard to the patient [38-40]. Kusnetzky and co-workers reported single-centre data on 18.671 consecutive studies and found no increased acute mortality in patients who had received a contrast agent [39]. Main et al. reported data from a multicenter registry that included 4.300.966 consecutive patients [38]. Their finding was that patients who had received echo contrast actually had a lower mortality rate compared to those who had not received contrast. Furthermore, Dolan et al. compared 23.659 patients from three U.S. medical centres who had received echo contrast, at a rest examination, with 5.900 controls who had not received contrast, and found no increased mortality or nonfatal myocardial infarct in patients who had received contrast [40]. Dolan and co-workers extended their analysis and compared 10.788 patients who had undergone stress echocardiography (DSE or exercise stress echocardiography) and received contrast with 15.989 who had not received contrast. No increased mortality or nonfatal myocardial infarct in patients who had received contrast could be found also in this cohort. These studies clearly show that using echo contrast in stable patients does not pose a significant risk. This knowledge must be weighted against the hazards of a non-diagnostic echocardiography examination, and the potential risks accompanied by alternative tests. The use of contrast in difficult to image patients has been proven cost effective [41], however cost effectiveness remains to be demonstrated in the setting of RTP-ASE.

## Conclusion

The results of the present agreement study indicate that RTP-ASE Qontrast^® ^quantification of myocardial ischaemia is less accurate than visual evaluation, as compared with SPECT. At present this method cannot be recommended for clinical use, but future development of echocardiographic image quality and software properties may improve the method.

## Competing interests

The authors declare that they have no competing interests.

## Authors' contributions

PG, RW, MD and RBW initiated the study. RW, MD and OT supervised the study and participated in the interpretation of the results and manuscript preparation. PG performed measurements, made all data conversions, plots and calculations from ultrasound data, and participated in the preparation of the manuscript. PG, KS, RW and MD participated in data collection, performed statistical analysis and participated in the interpretation of the results. LL and RBW participated in the interpretation of the results, in the creation of plots and in the preparation of the manuscript. MK participated in the interpretation of the results and preparation of the manuscript. All authors read and approved the final manuscript.
